# Radiomics Nomograms Based on Multi-Parametric MRI for Preoperative Differential Diagnosis of Malignant and Benign Sinonasal Tumors: A Two-Centre Study

**DOI:** 10.3389/fonc.2021.659905

**Published:** 2021-05-03

**Authors:** Shu-cheng Bi, Han Zhang, He-xiang Wang, Ya-qiong Ge, Peng Zhang, Zhen-chang Wang, Da-peng Hao

**Affiliations:** ^1^ Department of Radiology, The Affiliated Hospital of Qingdao University, Qingdao, China; ^2^ GE Healthcare (China), Shanghai, China; ^3^ Department of Radiology, Beijing Friendship Hospital, Capital Medical University, Beijing, China

**Keywords:** radiomics, magnetic resonance imaging, benign, malignant, sinonasal, differential diagnosis

## Abstract

**Objectives:**

To investigate the efficacy of multi-parametric MRI-based radiomics nomograms for preoperative distinction between benign and malignant sinonasal tumors.

**Methods:**

Data of 244 patients with sinonasal tumor (training set, n=192; test set, n=52) who had undergone pre-contrast MRI, and 101 patients who underwent post-contrast MRI (training set, n=74; test set, n=27) were retrospectively analyzed. Independent predictors of malignancy were identified and their performance were evaluated. Seven radiomics signatures (RSs) using maximum relevance minimum redundancy (mRMR), and the least absolute shrinkage selection operator (LASSO) algorithm were established. The radiomics nomograms, comprising the clinical model and the RS algorithms were built: one based on pre-contrast MRI (RNWOC); the other based on pre-contrast and post-contrast MRI (RNWC). The performances of the models were evaluated with area under the curve (AUC), calibration, and decision curve analysis (DCA) respectively.

**Results:**

The efficacy of the clinical model (AUC=0.81) of RNWC was higher than that of the model (AUC=0.76) of RNWOC in the test set. There was no significant difference in the AUC of radiomic algorithms in the test set. The RS-T1T2 (AUC=0.74) and RS-T1T2T1C (RSWC, AUC=0.81) achieved a good distinction efficacy in the test set. The RNWC and the RNWOC showed excellent distinction (AUC=0.89 and 0.82 respectively) in the test set. The DCA of the nomograms showed better clinical usefulness than the clinical models and radiomics signatures.

**Conclusions:**

The radiomics nomograms combining the clinical model and RS can be accurately, safely and efficiently used to distinguish between benign and malignant sinonasal tumors.

## Introduction

Benign and malignant tumors are widely distributed in the sinonasal area (1–3). Patients with malignant sinonasal tumors often require surgery, radiotherapy or chemotherapy, and usually have a poor prognosis (4), while patients with benign tumors usually require only clinical follow-up or direct total surgical resection (5, 6). Thus, it is important for the radiologist to differentiate between the types (7–9). In most cases, histopathological analysis is a safe and important diagnostic tool for the evaluation of sinonasal tumors (10). However, its diagnostic sensitivity is low because of the surrounding inflammatory tissue, which usually accompanies the biopsy specimen. CT and MRI imaging play a crucial role in the differentiation of sinonasal tumors (11–13). However, the image morphologies of malignant and benign sinonasal neoplasms often overlap, and are nonspecific (12, 14, 15). Hence, a non-invasive, accurate, reliable, and convenient method for distinguishing between benign and malignant sinonasal tumors is necessary.

Radiomics is an emerging method for medical image analysis (16). Radiomics is defined as a favorable biomarker which extracts and analyzes a large number of advanced quantitative features from medical imaging (17, 18). This method can identify the heterogeneity and microenvironment of various tumors. Recently, radiomics has been applied broadly to tumor qualitative analysis, evaluation of efficacy, genetic analysis, tumor staging, and prediction of prognosis (19–21). Additionally, radiomics is fast, economical, and reproducible. Therefore, MRI-based radiomics could be effective to distinguish malignant from benign sinonasal tumors.

However, to the best of our knowledge, radiomics has not been widely used to differentiated malignant from benign sinonasal tumors, and needs further research. The aim of this study was to evaluate the performance of the MRI-based radiomics nomograms in discriminating between benign and malignant sinonasal tumors.

## Materials and Methods

### Patients

We searched the medical data of patients with sinonasal tumors admitted at two institutions between March 2006 and April 2020. And the retrospective study was ethically approved by both hospital’s institutional review board and the need for written informed consent was waived off. The inclusion criteria were as follows: (a) patients with histopathologically confirmed sinonasal tumors; and (b) patients underwent MRI examination less than 10 days before surgery. And the exclusion criteria were as follows: (a) patients without complete medical data; and (b) Poor quality MRI images such as a signal-to-noise ratio(s/n) ≤1.0. In all, 244 patients with sinonasal tumor were retrospectively included in the research. 192 sinonasal tumors patients from our hospital constituted the training set, while 52 patients from another hospital constituted the test set according to the TRIPOD statement (22). According to the histopathological results and the latest WHO classification (23), all tumors were classified as benign or malignant tumor.

### MRI Image Acquisition

**Figure 1:**
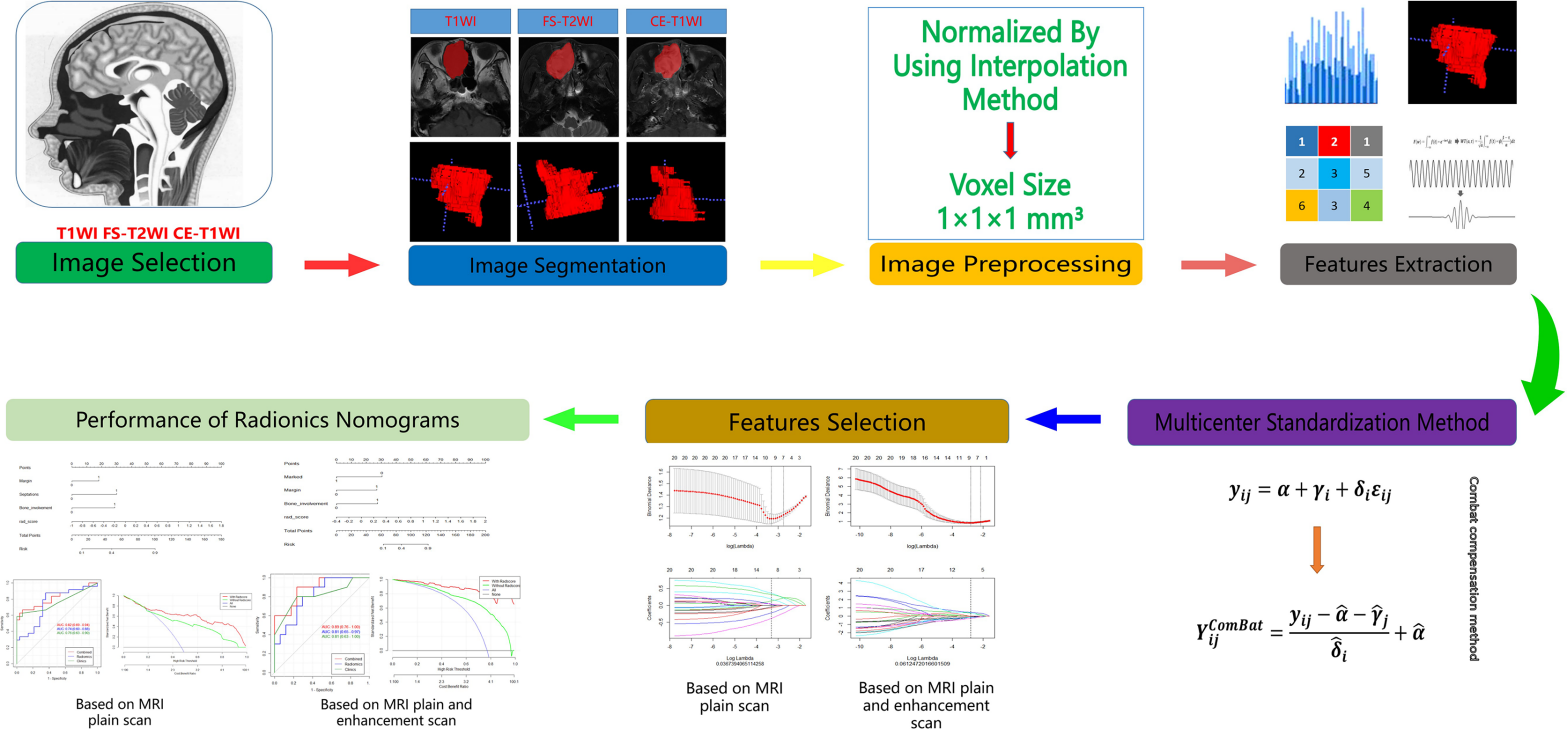
Flow chart of radiomics in this study.

All patients in this study underwent preoperative 1.5T and 3.0T MRI (GE Sigma, MagnetomTrio; GE Medical Systems Milwaukee WI, Siemens Healthineers). T1-weighted images (T1WIs) and fat-suppressed (FS) fast-spin-echo (FSE) T2-weighted images (T2WIs) were acquired. The 1.5T scanning parameters were as follows: T1WI (TR/TE, 400-600 ms/10-15 ms); FS-T2WI (TR/TE, 4000-4500 ms/120 ms); section thickness 4-5 mm; section spacing 0.4-0.5 mm; matrix 512×256; FOV 200×220 mm. The 3.0T scanning parameters were as follows: T1WI (TR/TE, 400-600 ms/10-15 ms); FS-T2WI (TR/TE, 3500-4500 ms/90-100 ms); section thickness 4-5 mm; section spacing 0.4-0.5 mm; matrix 512×256; FOV 200×220 mm. CE T1WIs required automatic intravenous injection of 0.2 mL/kg Gadopentetate Dimeglumine (Magnevist; Bayer Schering, Berlin, Germany). The radiomics work process is shown in **Figure 1**.

### MRI Image Processing and Radiomics Feature Extraction

By using ITK-SNAP software (http://www.itksnap.org) (24), three-dimensional region of interest (3D-ROI) was conducted on the preoperative T1WIs, FS-FSE-T2WIs, and CE-T1WIs. According to the contour of the tumor from each transverse layer, the ROI was outlined on preoperative T1WI, FS-FSE-T2WI, and CE-T1WIs sequences, and turned into a 3D-ROI automatically. The 3D-ROI segmentation included the entire primary tumor while avoided obvious peripheral inflammation, cystic or necrotic regions and macrovessels.

The intra and inter-observer performance during radiomics feature extraction was evaluated by calculating the intra-/inter-observer correlation coefficients (ICCs). Two radiologists with five years of working experience drew the 3D-ROIs, who were blinded to the clinical and pathologic information. And the same radiologists performed the second ROI manual segmentation after 1 month. Sinonasal tumors with both intra and inter-observer ICCs ≥0.80 were included in subsequent analysis. ICCs above 0.75 indicated good performance.

Feature extraction from medical images was executed on the preoperative T1WIs, FS-FSE-T2WIs, and CE-T1WIs by using the open-source Pyradiomics package (https://github.com/Radiomicss/pyradiomicss) (25). Many MRI texture features and the heterogeneity within the ROIs can be processed satisfactorily by the software. The MRI texture recognition is improved by using a series of preprocessing methods. First, all MRI images were resampled to voxel size of 1×1×1 mm³. Next, the MRI image intensity was normalized into standardized intensity with a mean value of zero and a standard deviation value of one. Ultimately, quantitative texture features were extracted with 1,224 totally. The seven categories of feature extraction were as follows: shape; grey level co-occurrence matrix; grey level run length matrix; grey level size zone matrix; grey level dependence matrix; first-order statistics and neighborhood grey tone difference matrix.

### ComBat Compensation Method

The maximum likelihood method was used to estimate additive and multiplicative batch effects based on a given feature distribution. Recent research showed its potential in improving the repeatability between different centers (26, 27). This study corrected the MRIner models by applying the R ComBat script (https://github.com/jfortin1/ComBatHarmonization) (28).

### Analysis of Morphological Features of MRI Image

All MRI images were assessed by two radiologists with ten years of working experience who distinguished malignant from benign tumors based on visual assessment, and were blinded to the clinical and pathologic information. The criteria of morphological MRI feature to evaluated sinonasal tumors are as follows: (1) heterogeneity; (2) T1 hyperintensity signal matrix; (3) T2 hypointensity signal matrix; (4) margin (well or ill- defined); (5) size (major axis <5 cm or ≥5 cm); (6) necrosis matrix; (7) myxoid matrix; (8) septations; (9) degree of enhancement (mild, moderate and marked); (10) pattern of enhancement (non-homogeneous) and (11) bone involvement (including osteosclerosis, bone destruction, or both). Presence of necrosis or myxoid matrices, and septations were defined as being beyond 10% of the entire tumor. The above MRI features were selected based on a previous research (29). MRI morphological features1−3 and 6−11 were categorized as positive or negative.

### Construction of Radiomics Signature

**Figure 2:**
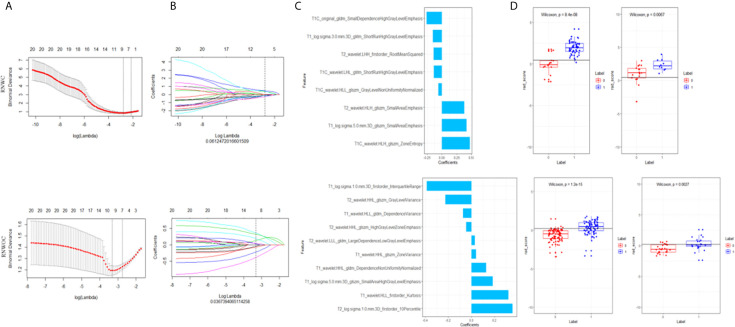
Selection of MRI features and confirmation of the predictive accuracy of RS. **(A)** Selection of the tuning parameter (λ). An optimal λ value of 0.061(RNWC)/0.037(RNWOC) with ln(λ)=–2.80/–3.30 was selected. **(B)** The coef-ficients have been plotted vs. ln(λ). **(C)** The selection of features with non-zero coeffi-cients and their corresponding roles. **(D)** The differential diagnostic efficacy of rad-scores.

R software (version 3.5.1) was applied to select MRI feature and construct radiomics model. First, the maximum relevance minimum redundancy (mRMR) algorithm was used to obtain the top 20 relevant features and eliminate the redundant and irrelevant feature for distinction between malignant and benign tumors. Next, the most predictive features were selected by the least absolute shrinkage and selection operator (LASSO) regression analysis. MRI features with non-zero coefficient variables were obtained and partial candidate feature coefficients were compressed to zero. Subsequently, a total of the seven radiomics signatures were constructed through the selected features of single sequences, combined sequences, and multi-parametric sequences as shown in **Figure 2**. The radiomics scores (rad-scores) of each patient were calculated. The performance of the radiomics signatures in distinguishing malignant from benign tumors were evaluated by the areas under the receiver operating characteristic (ROC) curve (AUC) of the test sets.

### Development of Clinical Model and Radiomics Nomogram

By using Wilcoxon’s test (continuous variables) and Chi-square-test (nominal variables), the clinical data and MRI morphological features were analyzed. Univariate logistic analysis was used to analyzed the features with p <0.1, and a multivariate logistic regression analysis was used to select the independent predictive factors for sinonasal malignancy by analyzing features with p <0.05. Factors with p <0.1 were considered significant and were used to create the clinical models. Finally, to establish reliable radiomics nomograms, the clinical models and the best RS were evaluated by multivariate logistic regression. Two different radiomics nomograms for this study were created (one based on pre-contrast MRI with CE T1WI, and another based on pre-contrast MRI).

### Performance Assessment of Different Models

To assess the goodness of fit of the radiomics nomograms, the Hosmer-Lemeshow (30) test was executed. The ability of the models to identify malignant and benign tumors was evaluated by comparing the following indicators in the training and test sets: AUC, accuracy, sensitivity, and specificity. The Delong test was used to analyze the AUC between each two of all the models. The reliability and effectiveness of the nomograms was evaluated by using a decision curve analysis (DCA). The performance of DCA was obtained by analyzing the net benefits of a series of threshold probabilities in the entire retrospective cohort (31).

### Statistical Analysis

All analytical methods in this study such as Chi-square-test, Wilcoxon’s test, Hosmer-Lemeshow test, LASSO regression, Delong test, ICC, ROC curve, calibration curve, and DCA were performed using R statistics software v.3.5.1 (https://www.Rproject.org). A p-value of <0.05 was considered statistically significant.

## Results

### Clinical Information and MRI Features of Patients

The diagnostic results and classification of the 244 patients are shown in **Supplementary S1**.

The clinical data and MRI morphology of patients with pre-contrast MRI are shown in **Table 1**. The results of p <0.10 were considered significant and were included in the subsequent study shown in **Table 3**. The independent predictors identified for sinonasal tumor malignancy were necrosis, margin, septations, and bone involvement (all p <0.05) based on the results of the univariate analyses. A clinical model involving bone involvement, margin, and septations (all p <0.10) in the test set (AUC=0.76) were established based on the results of the multivariate logistic regression analysis.

**Table 1:** Demographic **D**ata and **M**orphological **F**eatures of **P**recontrast MRI.

		Training set (n=192)			Test set (n=52)		
		malignant tumor (n=90)	benign tumor (n=102)	p-value	malignant tumor (n=28)	benign tumor (n=24)	P-value
Gender	male	59	69	0.7608	15	18	0.9037
	female	31	33		9	10	
Age (mean±SD)		54.56±15.88	54.56±15.51	0.6553	50.67±2.76	50.67±12.93	0.6329
T1 high signal	+	4	5	0.8841	3	6	0.4087
	–	86	97		21	22	
T2 low signal	+	6	10	0.4353	5	7	0.7342
	–	84	92		19	21	
Heterogeneous signal	+	69	67	0.096	18	17	0.2833
	–	21	35		6	11	
Size	≥5cm	39	33	0.1181	15	13	0.2555
	< 5cm	51	69		9	15	
Margin	Well-defined	34	76	< 0.0001	11		0.016
	Ill-defined	56	26		13	6	
Myxoid	+	77	76	0.0585	16	6	0.0501
	–	13	26		8	22	
Necrosis	+	22	9	0.0034	8	4	0.1103
	–	68	93		16	24	
Sepetations	+	29	7	<0.0001	13	10	0.1895
	–	61	95		11	18	
Bone involvement	+	58	18	<0.0001	15	4	<0.0001
	–	32	84		9	24	

The clinical and MRI morphology of patients who underwent pre-contrast and post-contrast MRI are shown in **Table 2**. The results of p <0.10 were considered significant and were included in the subsequent study shown in **Table 3**. The malignant predictive factors were bone involvement, heterogeneity, margin, and marked enhancement on MRI (all p <0.05) based on the results of univariate analyses. Based on the results of the multivariate logistic regression analysis, the parameters margin, bone involvement, and marked enhancement (all p <0.10) were included to create a clinical model, with an AUC of 0.81 in the test set. The performance of the clinical model based on pre-contrast and post-contrast MRI was higher than that of the clinical model based only on pre-contrast MRI.

**Table 2:** Demographic **D**ata and **M**orphological **F**eatures With MRI **E**nhancement.

		Training set (n=74)			Test set (n=27)		
		Malignant tumor (n=57	Benign tumor (n=17)	P-value	Malignant tumor (n=10)	Benign tumor (n=17)	P-value
Gender	male	38	14	0.2204	8	12	0.6200
	female	19	3		2	5	
Age (mean+SD)		56.05±15.31	56.07+16.10	0.7773	55.60+7.55	55.06+15.43	0.9399
T1 high signal	+	4	3	0.1961	0	1	0.4900
	–	53	14		10	16	
T2 low signal	+	0	4	0.0002	1	5	0.2649
	–	57	13		9	12	
Heterogeneous signal	+	48	8	0.0019	10	8	0.0062
	–	9	9		0	9	
Size	≥5cm	25	4	0.1364	9	8	0.0308
	< 5cm	32	13		1	9	
Margin	Well-defined	42	12	0.0010	6	5	0.1327
	Ill-defined	15	5		4	12	
Myxoid	+	51	12	0.0577	10	16	0.4900
	-	6	5		0	1	
Necrosis	+	16	3	0.3958	6	2	0.0102
	-	41	14		4	15	
Sepetations	+	21	2	0.0525	10	8	0.0062
	-	36	15		0	9	
Bone involvement	+	44	5	0.0003	8	4	0.0056
	-	13	12		2	13	
Pattern of enhancement	+	46	11	0.1745	10	12	0.0677
	–	11	6		0	5	
Degree of enhancement	mild	31	6	0.1723	2	5	0.6200
	moderate	18	3	0.2703	6	7	0.3688
	marked	9	8	0.0077	2	5	0.6200

**Table 3:** Positive **R**esults of **U**nivariate & **M**ultivariate **L**ogistic **R**egression **A**nalysis for **M**alignant **S**tatus in **S**inonasal **T**umors.

	Variables	Univariate		P-value	Multivariate analysis		P-value
		OR	95%CI		OR	95%CI	
Precontrast MRI	Heterogeneity	1.72	0.91-3.28	0.0964			
	Margin	4.81	2.63-9.04	<0.0001	2.44	1.13-5.31	0.0232
	Myxoid	2.03	0.98-4.35	0.0605			
	Necrosis	334	1.49-8.08	0.0047			
	Septations	645	2.80-16.85	<0.0001	2.71	0.90-8.78	0.0836
	Bone involvement	8.46	4.42-16.86	<0.0001	4.31	1.97-9.70	< 0.0001
	Rad score				4.12	2.40-7.67	< 0.0001
MRI plain and Enhancement scam	Marked enhancement	0.21	0.06-0.69	0.0103	0.10	0.01-1.07	0.0815
	Heterogeneity	6.00	1.85-20.47	0.0031			
	Margin	6.72	2.13-24.21	0.0018	7.89	0.91-151.30	0.0959
	Myxoid	3.54	089-13.80	0.0650			
	Septations	4.38	1.09-29.51	0.0655			
	Bone involvement	8.12	2.54-29.71	0.0007	7.74	0.93-104.19	0.0775
	Rad score				23.20	4.92-298.20	0.0017

### Performance of the MRI-Radiomics Signatures

**Figure 3:**
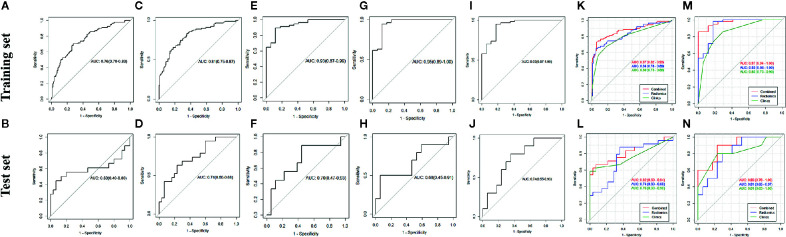
AUC of RS-T1 model **(A, B)**, RS-T2 model **(C, D)**, RS-T1C model **(E, F)**, RS-T1T1C model **(G, H)**, RS-T2T1C model **(I, J)**, Clinical model, RS-T1T2, RNWOC model **(K, L)** and Clinical model, RSWC, RNWC model **(M, N)** for distinguishing be-tween benign and malignant sinonasal tumors in the train set and test set.

AUCs of the seven algorithms are shown in **Figure 3**. In the training set, the following four algorithms had significant differences in AUC (RS-T1 and RS-T1C, p=0.0002; RS-T2 and RS-T1C, p=0.0038; RS-T1T1C and RS-T1T2, p=0.0087; RS-T2T1C and RS-T1T2, p=0.0304). There was no statistically significant difference between the AUC values of the remaining algorithms. There was no significant difference in the AUC of algorithms in the test set.

### Construction of the RNWOC and the RNWC and Performance of Different Models

**Figure 4:**
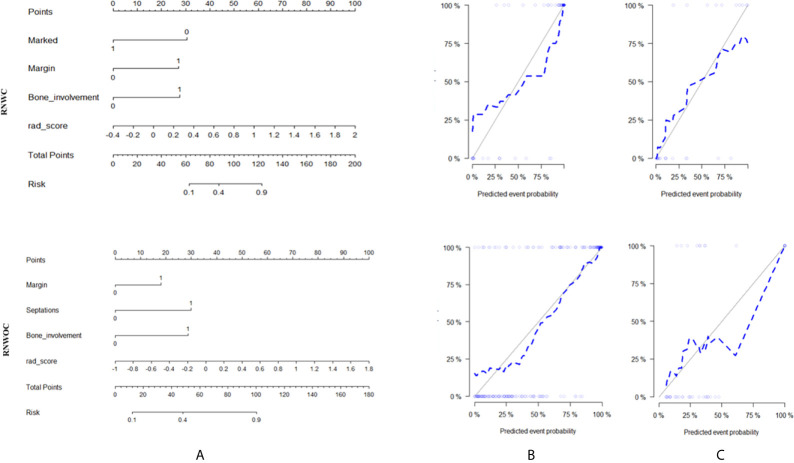
Radiomics nomograms **(A)**. Calibration curves of the radiomics nomograms in the training set **(B)** and test set **(C)**. The calibration curves showed that the nomograms had good agreement between the predictive risk of malignant status and the patho-logical outcome.

**Figure 5:**
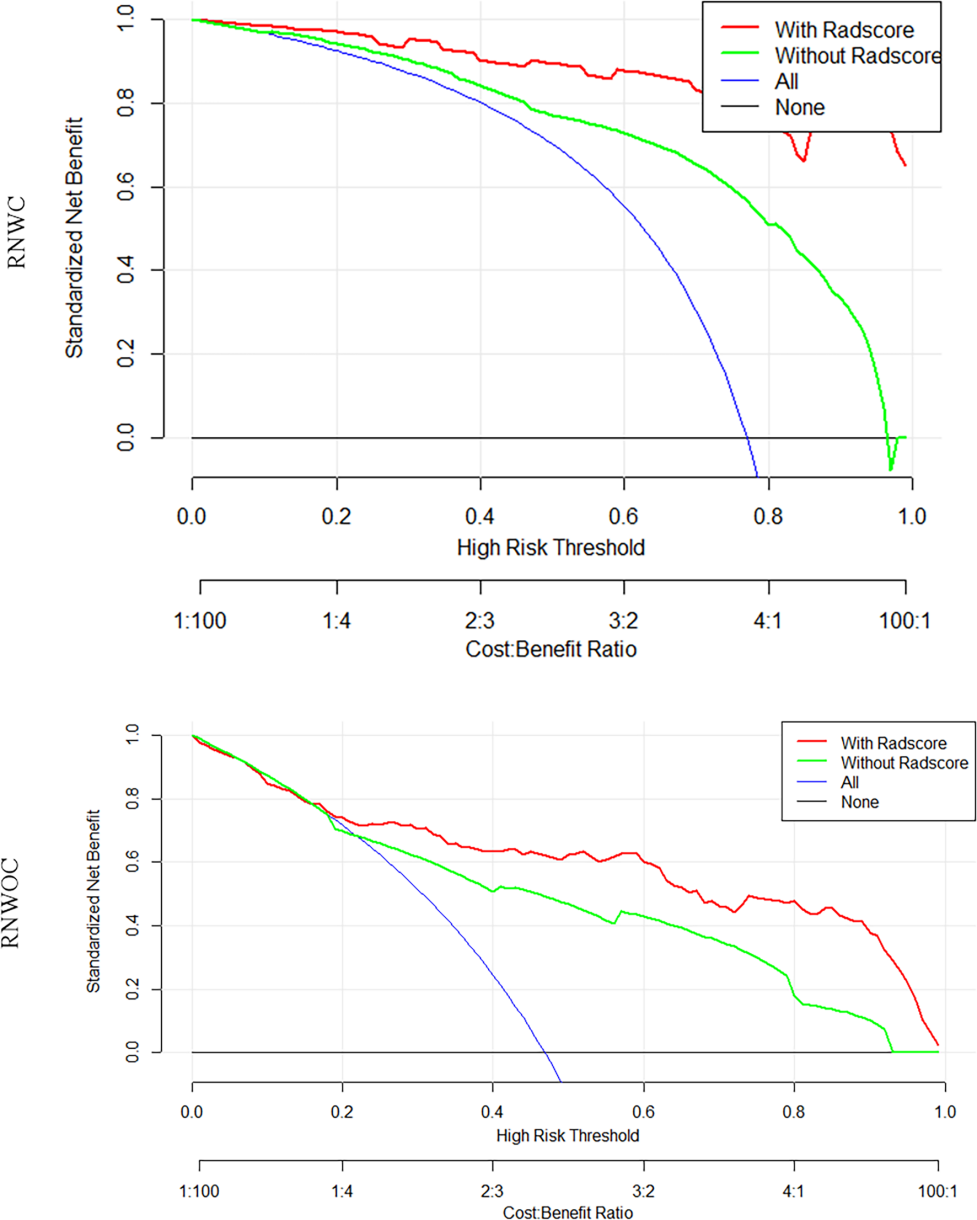
DCA of the radiomics nomograms. In the RNWC, the decision curves indicated that the radiomics nomograms were more beneficial than the clinical and RS model when the threshold probability is between 0.1 and 0.9. In the RNWOC, the threshold probability was between 0.2 and 1.0.

The RNWC and the RNWOC were subsequently constructed as shown in **Figure 4A**. RNWC was the combination of the RSWC algorithm and significant clinical factors based on pre-contrast and post-contrast MRI, while the RNWOC integrated the remarkable clinical factors based on pre-contrast MRI and the RS-T1T2 algorithm. The performance of the RNWC and the RNWOC is shown in **Tables 4** and **5**, respectively. The discrimination performance of the RNWC and the RNWOC was excellent in the test (AUC=0.89 and 0.82, respectively) sets. The calibration curves of the two nomograms indicated that the models were appropriate in both sets as shown in **Figure 4**. The DCA of the two nomograms are shown in **Figure 5**. The two nomograms showed better clinical usefulness than the clinical models and radiomics signatures.

**Table 4:** Results of **C**ombined **R**adiomics **N**omogram **P**redictive **A**bility for **D**istinguishing **B**etween **M**aligant and **B**enign **T**umors of **S**inonasal.

		Accurary	95%CI	Sensitivity	Specificity	PPV	NPV
RS-T1	Train Test	0.7157 0.7500	(0.6485-0.7765) (0.6040-0.8636)	0.7257 0.9333	0.7033 0.4444	0.7523 0.7368	0.6737 0.8000
RS-T2	Train	0.7291	(0.6624-0.7889)	0.6460	0.8333	0.8295	0.6522
	Test	0.7142	(0.5674-0.8342)	0.7667	0.6316	0.7667	0.6316
RS-T1T2	Train	0.8073	(0.7443-0.8605)	0.9510	0.6444	0.7519	0.9206
	Test	0.7500	(0.6105-0.8597)	0.6429	0.8750	0.8571	0.6774
clinical model	Train	0.7500	(0.6826-0.8096)	0.7561	0.7455	0.6889	0.8039
	Test	0.7885	(0.6530-0.8894)	0.8824	0.7429	0.6250	0.9286
RNWOC	Train	0.8438	(0.7845-0.8920)	0.9167	0.8000	0.7333	0.9412
	Test	0.8077	(0.6747-0.9037)	0.8889	0.8000	0.6667	0.9286

PPV, Positive predictive value; NPV, Negative predictive value.

**Table 5:** Results of **M**ulti-**P**arametric **R**adiomics **N**omogram **P**redictive **A**bility for **D**istinguishing **B**etween **M**alignant and **B**enign **T**umors of **S**inonasal.

		Accurary	95%CI	Sensitivity	Specificity	PPV	NPV
RS-T1C	Train	0.8919	(0.7980-0.9522)	0.8824	0.8000	0.7143	0.9623
	Test	0.6667	(0.4604-0.8348)	0.5556	0.8000	0.9091	0.5000
RS-T1T1C	Train	0.9324	(0.8493-0.9777)	0.8824	0.8000	0.8333	0.9643
	Test	0.7778	(0.5774-0.9138)	0.9412	0.8000	0.7619	0.8333
RS-T2T1C	Train	0.9189	(0.8318-0.9697)	0.8235	0.8000	0.8235	0.9474
	Test	0.7037	(0.4982-0.8625)	0.6471	0.8000	0.8462	0.5714
RSWC	Train	0.9459	(0.8673-0.9851)	0.8235	0.8000	0.9333	0.9492
	Test	0.7407	(0.5372-0.8889)	0.7059	0.8000	0.8571	0.6154
Clinical model	Train	0.8243	(0.7183-0.9030)	0.9074	0.8000	0.8596	0.7059
	Test	0.6667	(0.4604-0.8348)	0.5333	0.8000	0.8000	0.5882
RNWC	Train	0.8919	(0.7980-0.9522)	1.0000	0.8000	0.8596	1.0000
	Test	0.8148	(0.6192-0.9370)	0.6923	0.8000	0.9000	0.7647

PPV, Positive predictive value; NPV, Negative predictive value.

## Discussion

The aim of this study was to evaluate the performance of radiomics nomograms for preoperative differentiation between benign and malignant sinonasal tumors. We established two radiomics nomograms for preoperative identification of malignant sinonasal tumors. One was the RNWOC comprising the significant clinical factors based on pre-contrast MRI and RS-T1T2 algorithm. The other was RNWC, integrating the RSWC algorithm and significant clinical factors based on pre-contrast and post-contrast MRI that had favorable values for predicting malignant tumors. Compared to the RNWC, the ability of the RNWOC to identify malignant from benign sinonasal tumors had better generalization based more cases. However, the RNWC achieved relatively better efficacy than the RNWOC. Thus, the two radiomics nomograms can assist in clinical decision-making by preoperative identification of malignant sinonasal tumors.

There are different treatment regimens for benign and malignant sinonasal tumors (32, 33). Thus, distinction between benign and malignant tumors is essential for therapeutic decisions. In the present study, the morphological features based on the pre-contrast MRI (heterogeneity, T1 high signal, T2 low signal, size, margin, septations, myxoid, necrosis, and bone involvement) and the traditional clinical data (age and gender) were analyzed. In addition, the pattern of enhancement and degree of enhancement seen on the post-contrast MRI, were also analyzed. The results showed that margin, septations, bone involvement (on pre-contrast MRI) and marked enhancement, margin, bone involvement (pre-contrast and post-contrast MRI) are significant risk factors to predict malignant sinonasal tumors. However, the significant risk factors based on pre-contrast MRI achieved an AUC of 0.76 in the test set, whereas those based on pre-contrast and post-contrast MRI achieved an AUC of 0.81 in the test set. The clinical model based on pre-contrast and post-contrast MRI was relatively poor at effectively identifying benign and malignant tumors with 67% accuracy in the test set, while the clinical model based on pre-contrast MRI had an accuracy of 78%. The discriminating ability between these two models is different and unstable. The results might imply that contribution of the traditional clinical data and MRI morphology in the differential diagnosis of malignant and benign sinonasal tumors is limited.

In recent times, radiomics had surpassed the traditional visual assessment of CT and MRI images, and has become an effective and reliable image processing method that evaluates both biological and histological characteristics of lesions. Previous studies showed that radiomics had a strong ability to identify malignant from benign tumors. Zhang et al. (34) reported that the model based on radiomics features from TWI, DKI, and quantitative DCE pharmacokinetic parameter maps was a good tool to differentiate malignant and benign breast lesions, with an AUC of 0.92 in the test set. Wang et al. (35) reported that the radiomics nomogram can be used to classify between malignant and benign soft-tissue masses in the extremities with an AUC of 0.94 in the test set. This study analyzed all 1,224 MRI features to establish the radiomics using the mRMR and LASSO algorithm. The mRMR was reliable for the selection of features with more credible coefficients and fewer redundancies (36). LASSO can obtain the reliable features selected in both, ridge regression and subset, and is excellent for feature screening (37). Subsequently, seven radiomics algorithms were built to differentiate between benign and malignant sinonasal tumors. In these algorithms based on the single parametric MRI, we found that RS-T1C had the strongest ability to distinguish benign and malignant tumors. In algorithms based on multi-parametric MRI, RSWC had the best test efficacy. In addition, the accuracy of RSWC was higher than that of RS-T1C in differentiating between benign and malignant sinonasal masses in test set. The results demonstrated that multi-parameter MRI had a higher potential to reflect all information about the tumors, compared to the single-parameter MRI.

The MRI plays an important role in distinguishing between benign and malignant tumors in the sinonasal area. The RNWOC based on pre-contrast MRI images and RNWC based on pre-contrast and post-contrast MRI images were constructed. Depending on the perfusion and permeability of tumor blood vessels, the MRI enhanced images had a higher potential to reflect all tumor information compared to the pre-contrast MRI images. Thus, the performance of RNWC in differentiating between malignant and benign tumors in the sinonasal area was better than that of RNWOC. However, there are some limitations of post-contrast MRI that restricts its widespread use in clinical practice. Post-contrast MRI is an invasive procedure. Moreover, contrast agents might cause adverse reactions in some patients, aggravate the metabolic burden of liver and kidney, and be deposited in the brain leading to damage. Hence, the RNWOC based on pre-contrast MRI is widely used in clinical practice. To identify malignant from benign sinonasal tumors more accurately, the RNWC were also available. Further research is necessary to understand which strategy should be implemented.

By fully analyzing and using the clinical and imaging information, tumors in the sinonasal area can be diagnosed and treated more effectively. The MRI morphology and radiomics nomograms are two completely different imaging analysis and processing methods, but both are derived from imaging images. There is no standardization for the evaluation of MRI images, and the interpretation is subjective, based on the experience of the radiologist. However, in routine clinical practice, the identification malignant from benign sinonasal tumors by the radiologists is convenient and cost effective. Nevertheless, radiomics has significant advantages over the traditional image interpretation methods. The results of radiomics nomograms are quantifiable and independent of subjective factors. Moreover, radiomics nomograms can detect microscopic features such as tumor heterogeneity, which might be missed by the human eye. These advantages are significant in differentiating between benign and malignant sinonasal tumors.

A lot of research has been performed to distinguish benign from malignant sinonasal tumors. Wang et al. (38) reported that semi-quantitative DCE-MRI parameter was an effective method to identify malignant and benign neoplasms with an AUC of 0.693 and accuracy of 70.4%. Xiao et al. (39) found that the apparent diffusion coefficient (ADC) had a good performance in differentiating between benign and malignant tumors with AUC of 0.754 and accuracy of 68.6%. Zhang et al. (40) analyzed clinical parameters and MRI-based radiomics features of 197 sinonasal tumor patients, and found that the radiomic nomogram with an AUC of 0.91 can effectively predict malignant sinonasal tumors. The research results of this study are basically consistent with previously published relevant research results. Being different from previous studies, more cases, more quantitative radiomic features and post-contrast MR sequences are involved in our research.

## Limitations

There were several limitations in this study. First, the potential selection bias was inevitable since it was retrospective study. Second, the segmentation of ROIs in this study was by manual delineation, and thus could be vulnerable to potential human error. Third, this sample was relatively small, though we included cases from two medical institutions. Future studies with larger sample size are necessary. Fourth, this study has the potential to help in discriminating between benign and malignant sinonasal tumors. However, the image morphologies of malignant and benign sinonasal neoplasms often overlap. Radiomics would help in identifying malignant sinonasal tumor in this condition. And the final diagnosis is still requiring histopathology for confirmation. Further, the combat compensation method was used to preserve the distinguishing characteristics of texture patterns while eliminating influences of the scanner and protocol in this study. This strategy is appropriate to facilitate multicenter radiomic analyses. We will collect more MRI sequences, such as DCE-MRI or ADC, to verify the effectiveness of the method in a future study.

## Conclusion

In conclusion, we constructed and evaluated two radiomics nomograms in this study, which can effectively distinguish malignant from benign tumors in the sinonasal area. The RNWC had the best performance in differentiating between benign and malignant tumors. The RNWOC is widely used, simple and safe, and has high stability and reliability. We recommend these two models to be used in clinical practice.

## Data Availability Statement

The raw data supporting the conclusions of this article will be made available by the authors, without undue reservation.

## Ethics Statement

The studies involving human participants were reviewed and approved by The Affiliated Hospital of Qingdao University and Beijing Friendship Hospital, Capital Medical University. The ethics committee waived the requirement of written informed consent for participation.

## Author Contributions

S-CB and HZ have contributed equally to this work. D-PH and H-XW conceptualized the study. Y-QG, H-XW, and D-PH contributed to the methodology and validated the study. PZ, S-CB and HZ contributed to the formal analysis and investigation. S-CB and HZ wrote and prepared the original draft. All authors wrote and prepared the original draft. D-PH and Z-CW supervised the study. All authors contributed to the article and approved the submitted version.

## Conflict of Interest

Author Y-QG was employed by company GE Healthcare China.

The remaining authors declare that the research was conducted in the absence of any commercial or financial relationships that could be construed as a potential conflict of interest.

## Supplementary Material

The Supplementary Material for this article can be found online at: https://www.frontiersin.org/articles/10.3389/fonc.2021.659905/full#supplementary-material
