# Supplementary material for: Radiomics Nomograms Based on Multi-Parametric MRI for Preoperative Differential Diagnosis of Malignant and Benign Sinonasal Tumors: A Two-Centre Study
Source: Front Oncol. 2021 May 3;11:659905. doi: 10.3389/fonc.2021.659905 (PMC8127839; doi:10.3389/fonc.2021.659905)
Supplement: Supplementary file 1 [file Table_1.docx]

**Training**

**（**

**n=102)**

**External**

**validation**

**（**

**n=28**

**）**

**Training**

**（**

**n=90**

**）**

**External**

**validation**

**（**

**n=24**

**）**

Respiratory epithelial

adenomatoid hamartoma (REAH)

3

1

Adenoid cystic carcinoma

3

1

Hemangioma

10

3

Chondrosarcoma

2

1

Granuloma

2

0

Fibrosarcoma

5

0

Sinonasal papillomas

76

22

Lymphoma

9

4

Ossifying fibroma

2

1

Leiomyosarcoma

1

0

Plemorphic adenoma

2

0

Mucosal melanoma

5

2

Schwannoma

2

0

Neuroendocrine carcinoma

9

1

Neurofibroma

1

0

Olfactory neuroblastoma

5

0

Solitary fibroma

1

1

Rhabdomyosarcoma

3

1

Capillary endothelioma

2

0

Squamous cell carcinoma

44

12

Osteoma

1

0

Plasmacytoma

2

1

Ewing's sarcoma/

peripheralprimitive

neuroectodermal tumor(PNET)

2

1

**Benign tumor (n=130)**

**Malignant tumor (n=114)**

**S1. Summary of 244 Sinonasal Tumors Confirmed by Histologic Results**
